# Adenoviral vaccine targeting multiple neoantigens as strategy to eradicate large tumors combined with checkpoint blockade

**DOI:** 10.1038/s41467-019-10594-2

**Published:** 2019-06-19

**Authors:** Anna Morena D’Alise, Guido Leoni, Gabriella Cotugno, Fulvia Troise, Francesca Langone, Imma Fichera, Maria De Lucia, Lidia Avalle, Rosa Vitale, Adriano Leuzzi, Veronica Bignone, Elena Di Matteo, Fabio Giovanni Tucci, Valeria Poli, Armin Lahm, Maria Teresa Catanese, Antonella Folgori, Stefano Colloca, Alfredo Nicosia, Elisa Scarselli

**Affiliations:** 1 Nouscom Srl, Via Castel Romano 100, 00128 Rome, Italy; 2 Department of Molecular Biotechnology and Health Sciences, University of Turin, Via Nizza 52, 10126 Turin, Italy; 3 Nouscom AG, Bäumleingasse, 18 CH-4051, Basel, Switzerland; 4 Department of Molecular Medicine and Medical Biotechnology, University of Naples Federico II, Via Pansini 5, 80131 Naples, Italy; 5 CEINGE, Via Comunale Margherita, 484-538, 80131 Naples, Italy

**Keywords:** Cancer therapy, Cancer immunotherapy, Tumour vaccines

## Abstract

Neoantigens (nAgs) are promising tumor antigens for cancer vaccination with the potential of inducing robust and selective T cell responses. Genetic vaccines based on Adenoviruses derived from non-human Great Apes (GAd) elicit strong and effective T cell-mediated immunity in humans. Here, we investigate for the first time the potency and efficacy of a novel GAd encoding multiple neoantigens. Prophylactic or early therapeutic vaccination with GAd efficiently control tumor growth in mice. In contrast, combination of the vaccine with checkpoint inhibitors is required to eradicate large tumors. Gene expression profile of tumors in regression shows abundance of activated tumor infiltrating T cells with a more diversified TCR repertoire in animals treated with GAd and anti-PD1 compared to anti-PD1. Data suggest that effectiveness of vaccination in the presence of high tumor burden correlates with the breadth of nAgs-specific T cells and requires concomitant reversal of tumor suppression by checkpoint blockade.

## Abstract (web summary)

Vaccination against neo-antigens has resulted in an effective antitumor response in several models. Here, the authors show that delivery of larger sets of neo-antigens using an adenovirus-based vaccination platform, results in much better tumor protection when combined with checkpoint blockade in a mouse model of advanced disease.

## Introduction

Therapeutic efficacy of cancer vaccination depends on several factors, including: (i) the selection of the most appropriate target tumor antigens (ii) the ability to counteract the immune-suppressive tumor microenvironment, and (iii) the efficiency of the vaccination platform at inducing robust cellular immunity.

Regarding the antigen choice, neoantigens (nAgs) are emerging as an attractive target for cancer vaccines. nAgs are expressed in tumor cells as a result of mutations occurring in coding genes. They are non-self-peptides, without pre-existing central tolerance, with the potential of inducing strong immunogenicity and effective antitumor activity compared with classical tumor-associated antigens represented by overexpressed self-proteins^1,2^. As nAgs are unique to cancer cells, the risk of inducing autoimmunity is very low, qualifying them as safe immunogens with low risk of damaging normal healthy tissues. Evidence on the relevance of nAgs for the success of immunotherapy comes from the striking correlation found between tumor mutational burden and the efficacy of checkpoint blockade (CPB)^3,4^.

Moreover, preclinical studies showed effectiveness of nAgs cancer vaccination in several models, prompting initiation of clinical testing of nAgs-based vaccines by means of different approaches, including peptides and RNA^5–7^. Results from phase-I clinical studies showed a good safety profile and induction of immune responses, mainly CD4^+^ T cells, against some nAgs^8–10^.

Cancer cells promote their survival by limiting host immune response through several mechanisms. PD1/PDL1 interaction has been shown to have a key role in the impairment of anticancer T-cell immunity^11,12^. Therapeutic treatment with antibodies blocking the interaction between PD1 and PDL1 results in clinical benefit due to their ability to reverse the “exhausted” phenotype of spontaneously induced antitumor T cells^13–17^. Despite their success in many advanced malignancies, PD1/PDL1 blockade is efficacious only in a minority of treated patients^18^. nAgs-based vaccination is expected to synergize with CPB or other immunomodulatory drugs, able to revert tumor-induced immunosuppression, by increasing the breadth and potency of nAgs-specific T cells.

A paradigm for an efficient induction of cytotoxic T cells is that the antigen is expressed from within the cells. This requirement makes viral vectored vaccines one of the preferred technologies for the induction of effective antitumor immunity. Adenoviruses have been shown to be a powerful genetic vaccine platform. In particular, non-human Great Apes-derived Adenoviruses (GAd) represent an optimal choice with respect to the widely used human Adenovirus 5 (hAd5) because they can overcome the issue of anti-Ad5 pre-existing immunity present in humans that negatively affects its potency as a vaccine vector^19^. Indeed, a large number of clinical studies have shown potent immunogenicity and very good safety and tolerability of GAd vectors derived from different species (i.e., Chimpanzees and Bonobos)^20–25^.

One unique feature of the GAd platform is the ability to encode for large antigens (ie, over 2000 amino acids), as for a candidate HCV vaccine in phase 2 of clinical development^23^. Moreover, GAd-encoding artificial antigens generated by joining several fragments from different proteins led to unprecedented T-cell responses in humans^26^. These characteristics make GAd vectors particularly suitable to encode large artificial antigens composed of strings of nAgs peptides derived from patient-specific tumor mutations. In this study, to validate GAd as an effective and novel neoantigen-based cancer vaccine, we have measured the antitumor activity in several settings of low and high tumor burden. We demonstrate that effective antitumor response in the presence of high tumor burden requires concomitant treatment of GAd vaccine with immunomodulatory molecules able to counteract the immune-suppressive tumor microenvironment, such as anti-PD1 or anti-PDL1 antibodies. The contribution of the vaccine to the CPB therapy analyzed by transcriptomic analysis demonstrates diversification of the intratumoral T-cell repertoire in animals treated with GAd vaccine and anti-PD1. The translational relevance of our results is supported by the observation that mice responding to the treatments show upregulation of genes linked to T-cell activation and effector functions, including those belonging to a tumor inflammation signature (TIS) found in patients responding to anti-PD1 immunotherapy^27,28^.

## Results

### GAd induces potent neoantigen-specific T-cell responses

**Fig. 1:**
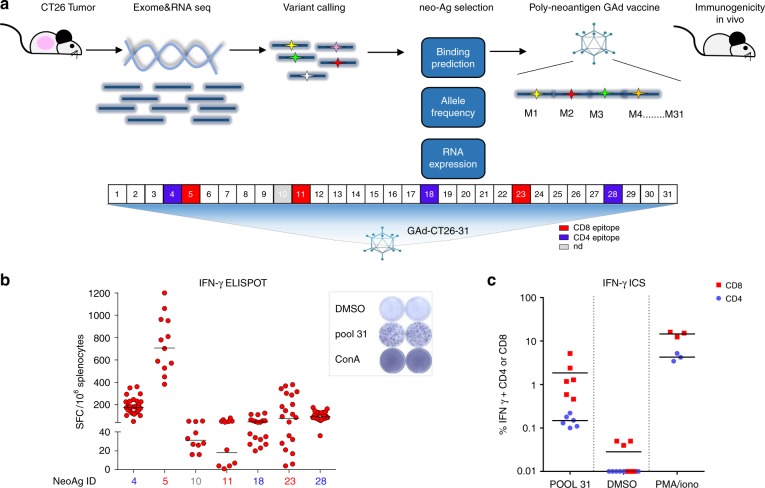
In vivo immunogenicity of GAd encoding CT26 neoantigens. **a** Schematic of the approach used to identify CT26 tumor specific mutations and generation of the vaccine; analysis of non-synonymous single nucleotide variants on DNA and RNA NGS allowed the selection of 31 nAgs, which were prioritized according to (i) MHC class I (predicted IC50 ≤ 500 nm) and II (binding score ≤ 1) binding predictions, (ii) tumor allele frequency (MAF ≥ 25%), and (iii) RNA expression (≥1 mutated RNA read). Selected nAgs were cloned in tandem in a GAd vector and tested in vivo. **b** In vivo immunogenicity of GAd-CT26-31. T-cell responses were measured by IFN-γ ELISpot on splenocytes of naive mice 3 weeks post immunization with 5 × 10^8^vp of GAd-CT26-31 (*n* = 10–30 mice/group). Responses against individual nAgs peptides found immunogenic are shown; nAgs ID is in red for nAgs inducing CD8^+^ T-cell responses or in blue for nAgs inducing CD4^+^ T-cell responses. Peptide diluent DMSO and Concanavalin A were used as negative and positive control, respectively. Data are representative of three independent experiments. **c** The quality of induced T-cell responses (CD4, blue circles, and CD8, red squares) was assessed by IFN-γ ICS by using a pool of 31 nAgs peptides (POOL 31) (*n* = 6 mice/group, representative of three experiments). Peptide diluent DMSO and PMA/Ionomycin were used as negative and positive control, respectively. SFC = Spot forming cells, ConA = Concanavalin A

We selected the murine colon carcinoma CT26 cell line because of its high nAgs load and responsiveness to anti-PD1 therapy^29,30^. CT26 were injected subcutaneously in mice and, once the tumors developed, they were harvested and processed for DNA and RNA sequencing by next-generation sequencing (NGS). Candidate nAgs were selected from the identified mutations based on: (i) MHC-I and II predictions of binding affinity^31^, (ii) mutation allele frequency in tumor DNA, and (iii) mRNA expression. Thirty-one single-nucleotide variant (SNV) mutations were prioritized based on the sequential use of specific cut-off values for each of the three parameters (Fig. 1a and Table 1). Each amino acid (aa) change determined by a SNV was flanked upstream and downstream by 12 wild-type aa for a total length of 25 aa in order to generate a nAg containing the maximum number of potential CD4^+^ and/or CD8^+^ T-cell epitopes. The 31 nAgs selected by this process were joined head to tail to generate a single artificial protein and the corresponding gene was cloned in a GAd vector (GAd-CT26-31), taking advantage of its large cloning capacity. Single injection of the vector induced potent T-cell immunity in naive mice as measured by ex vivo IFN-γ ELISpot. Seven out of 31 predicted neoantigens were able to elicit a detectable immune response (Fig. 1b), with induction of robust CD8^+^ and CD4^+^ T-cell responses measured on the pool of 31 peptides encoded by the vaccine (Fig. 1c). The quality of T-cell responses induced by individual nAgs was determined by intracellular cytokine staining (ICS) and fluorescence-activated cell sorting (FACS) analyses for six out of seven immunogenic nAgs. Three nAgs induced CD4^+^ T cells and three were able to induce CD8^+^ T-cell responses (Supplementary Fig. 1), showing induction of a well-balanced CTL response. Notably, cross-reactive T-cell responses against the corresponding wild-type peptides were not detected (Supplementary Fig. 2a).

**Table 1:** CT26 neoantigens encoded by GAd-CT26-31 vaccine

ID	Gene	Neoantigen sequence	RNA reads (mut/wt)	MAF (%)	MHC-I IC50	MHC-II score
1	Phf3	PGPQNFPPQNMF[G/E]FPPHLSPPLLPP	6/10	57	**41.5**	48.7
2	Zeb1	GAQEEPQVEPLD[L/F]SLPKQQGELLER	8/1	36	**121.0**	47.5
3	Trappc12	AVFAGSDDPFAT[A/P]LSMSEMDRRNDA	5/21	26	**457.9**	12.7
4	Aldh18a1	HSGQNHLKEMAI[P/S]VLEARACAAAGQ	12/7	51	13035.9	**0.05**
5	E2f8	ILPQAPSGPSYA[I/T]YLQPAQAQMLTP	3/5	32	**320.5**	6.8
6	Gid8	MSYAEK[P/S]DEITKDEWMEKL	7/31	26	**67.0**	49.8
7	Hdac2	GAGKGKYYAVNF[P/S]MRDGIDDESYGQ	29/30	40	**39.48**	40.5
8	Ttl	YRGADKLCRKAS[L/S]VKLVKTSPELSE	4/8	46	4768.9	**0.2**
9	Haus6	DSNLQARLTSYE[A/T]LKKSLSKIREES	29/9	33	**18.2**	7.49
10	Ndc1	HSFIHAAMGMAV[A/T]WCAAIMTKGQYS	3/1	70	15203.7	**0.84**
11	Glud1	LRTAAYVNAIEK[V/I]FKVYNEAGVTFT	353/89	68	**141.15**	9.4
12	G2e3	FEGSLAKNLSLN[S/F]QAVKENLYYEVG	12/15	53	**467.9**	21.1
13	Cad	DPRAAYFRQAEN[G/D]MYIRMALLATVL	11/3	52	2660.3	**0.7**
14	Dars2	LRSQMVMKMREY[L/F]CNLHGFVDIETP	6/7	46	3795.6	**0.39**
15	Smarcd1	DLLAFERKLDQT[I/V]MRKRLDIQEALK	16/2	46	11015.8	**0.99**
16	Zfp955b	IKREKCWKD[V/A]TY[S/P]ESFHTLESVPAT	2/16	45	**271.8**	9.2
17	Rwdd2b	GRSSQVYFTINV[S/N]LDLSEAAVVTFS	2/9	45	**143.0**	8.6
18	Slc20a1	KPLRRNNSYTSY[T/I]MAICGMPLDSFR	50/47	42	**118.0**	3.5
19	Ddx27	TTCLAVGGLDVK[S/F]QEAALRAAPDIL	13/16	41	8042.7	**0.6**
20	Top3a	IYEFDYHLYGQN[V/I]TMIMTSVSGHLL	1/1	41	**124.9**	11.4
21	Slc41a2	PDSFSIPYLTAL[G/D]DLLGTALLALSF	2/5	40	**134.8**	24.5
22	Ttc39a	YATILEMQAMMT[F/L]DPQDILLAGNMM	8/0	37	4108.0	**0.2**
23	Mtch1	SWIHCWKYLSVQ[G/S]SQLFRGSSLLFRR	160/226	36	**13.46**	2.2
24	Suv39h2	YDNKGITYLFDL[D/Y]YESDEFTVDAAR	2/7	36	**344.48**	33.6
25	Tomm70a	AQAAKNKGNKYF[K/Q]AGKYEQAIQCYT	17/41	35	**459.4**	5.2
26	Csnk2b	QPMLPIGLSDIP[G/D]EAMVKLYCPKCM	79/146	34	**202.2**	11.8
27	Caprin2	HRGAIYGSSWKY[S/F]TFSGYLLYQD	1/1	31	**36.8**	50.7
28	Dhx35	VIQTSKYYMRDV[T/I]AIESAWLLELAP	5/11	29	**67.5**	**0.7**
29	Xpot	PRGVDLYLRILM[A/P]IDSELVDRDVVH	17/53	28	**41.5**	4.7
30	Dclre1c	QIEQDALCPQDT[H/Y]CDLKSRAEVNGA	6/6	40	**69.1**	26.3
31	Noc3l	ALASAILSDPES[H/Y]IKKLKELRSMLM	5/3	60	**101.2**	10.7

List of CT26 neoantigens encoded by GAd-CT26-31 vaccine. nAgs were selected according to (i) MHC class I (predicted IC50 ≤ 500 nm) and MHC class II (binding score ≤1) binding predictions, (ii) tumor allele frequency (MAF ≥ 25%), and (iii) RNA expression (≥1 mutated RNA read). The mutated amino acid is underlined. Predicted binders to MHC-I and II are highlighted in bold. MAF = minor allele frequency

### Early vaccination with GAd controls tumor growth

**Fig. 2:**
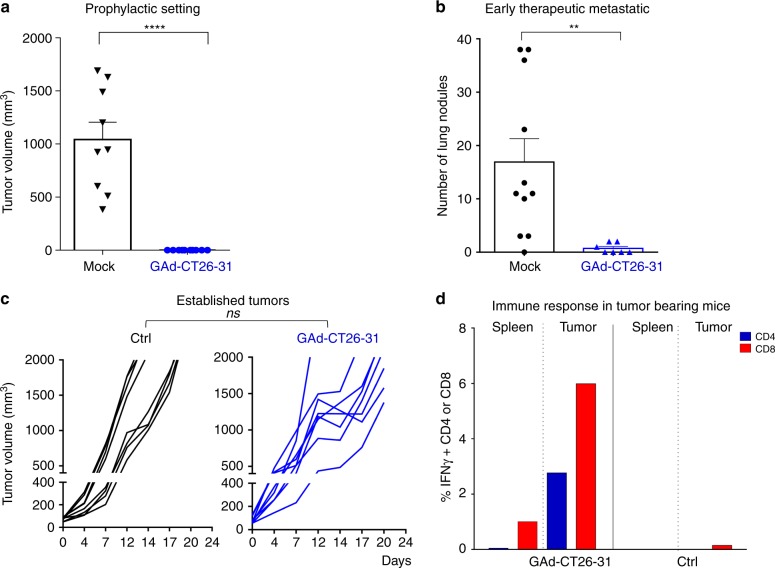
Early vaccination with GAd effectively controls tumor growth. **a** Mice (*n* = 8–10/group) were vaccinated with GAd-CT26-31; 2 weeks after immunization, CT26 cells were injected s.c. and tumor growth was monitored over time. Tumor volume measured 28 days post inoculation in GAd versus untreated (mock) mice is shown (two-tailed Mann–Whitney *U* test; ****p < 0.0001). **b** Mice (*n* = 8–10/group) were inoculated i.v. with CT26 cells (day 0) and left untreated (mock) or vaccinated with GAd-CT26-31 at day 3. The number of lung nodules counted at day 16 is shown (two-tailed Mann–Whitney *U* test; **p < 0.01). **c** Treatment with GAd vaccine started at day 0, on mice randomized according to tumor volume (mean 70–100 mm^3^, *n* = 8/group). Tumor volumes determined over time for individual tumors are shown. (n.s., not significant by two-tailed Mann–Whitney *U* test). **d** For analysis of immune responses in mice with established tumors, TIL or splenocytes were isolated from untreated or GAd-CT26-31-vaccinated groups and pooled (*n* = 4). The percentages of IFN-γ^+^ CD4^+^ or CD8^+^ T cells measured upon peptides pool re-stimulation are shown. Data from **a** to **d** are representative of 2–3 independent experiments

To investigate the effectiveness of our GAd nAgs-based vaccine in vivo, we tested its antitumor effect in the following early versus late vaccination settings: (1) prophylactic setting; (2) early intervention in a lung metastases model; (3) advanced therapeutic setting in large established subcutaneous tumors. In the first model, mice were first immunized with GAd-CT26-31 and subsequently challenged with CT26 tumor cells to evaluate the preventative value of the vaccination. This prophylactic intervention led to protection in 100% of vaccinated mice, whereas all untreated mice developed large tumors (Fig. 2a). Mice immunized with the same dose of a GAd vector encoding an unrelated antigen were indistinguishable from untreated mice (Supplementary Fig. 3). Similarly, GAd-CT26-31 was highly effective in eradicating lung metastases of CT26 cells in an early therapeutic setting, in which the vaccination was performed 3 days after intravenous injection of tumor cells (Fig. 2b).

We also generated a GAd vector (GAd-CT26-5) encoding the very same five CT26 nAgs previously published^7^, and we measured immunogenicity and efficacy in prophylactic and early treatment settings. Three of the five previously identified CT26 neoantigens (#5, #18, and #28) were found immunogenic post vaccination with GAd-CT26-5 (Supplementary Table 1 and Supplementary Fig. 4a). Interestingly, those three nAgs were also selected by our pipeline for inclusion in the longer GAd-CT26-31 construct. Vaccination with GAd-CT26-5 had also a potent antitumor activity in 100% of treated mice in both prophylactic and early treatment settings (Supplementary Fig. 4b–c).

Despite the efficacy observed in the prophylactic and early vaccination treatments, no antitumor activity was observed in mice bearing large established subcutaneous tumors vaccinated with either the GAd-CT26-31 (Fig. 2c) or the GAd-CT26-5 vector (Supplementary Fig. 4d).

To gain insights into the mechanism of tumor resistance, we measured vaccine-induced nAgs-specific T-cell responses in mice bearing large tumors. Despite the lack of efficacy in this setting, vaccine-induced nAgs-specific T cells were recovered from tumor infiltrates 10 days post vaccination and their effector function was measured ex vivo after re-stimulation with cognate nAgs peptides showing an effective production of IFN-γ by ICS assay (Fig. 2d). We also investigated the presence of spontaneous immune response against a previously described CD8^+^ T-cell epitope, gp70-AH1, derived from endogenous murine leukemia virus present in CT26 cells^32^. CD8^+^ T cells against gp70-AH1 were detected in untreated and vaccinated mice at similar levels (Supplementary Fig. 5). Thus, both spontaneous and vaccine-induced T cells can infiltrate tumors but cannot control growth of established tumors.

### Treatment of large tumors requires combination therapy

**Fig. 3:**
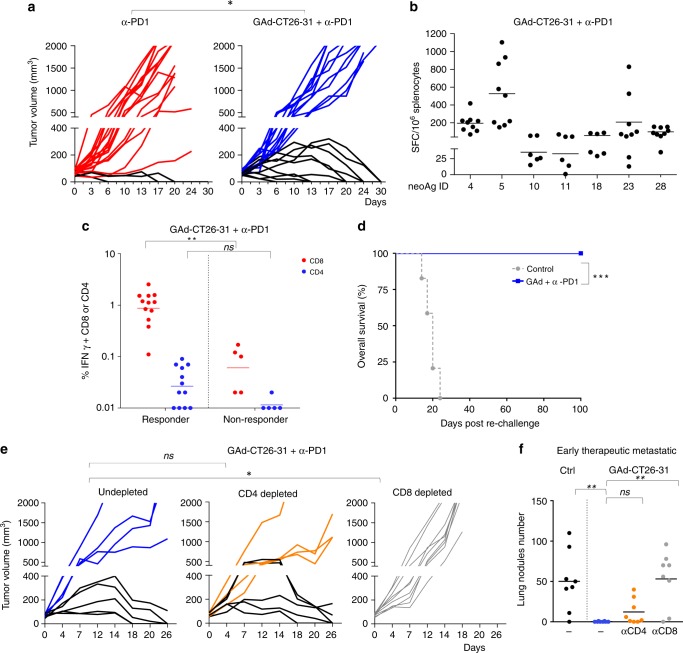
Efficacy of GAd in animals with high tumor burden requires the combination with anti-PD1. **a** Mice (*n* = 20/group) were inoculated s.c. with CT26 cells. One week later, animals were randomized according to tumor volume and treated with anti-PD1 alone (left panel) or in combination with GAd-CT26-31 (right panel). Vaccine was administered at day 0 (i.m.), whereas anti-PD1 was given twice per week until day 16 (i.p.). Tumor growth over time is shown for individual mice. Red and blue curves represent non-responder mice, black curves indicate responder mice. Data are from three independent experiments (two-tailed Chi-Square test; *p < 0.05). **b** Analysis of nAg-specific T-cell responses quantified by IFN-γ ELISpot in responder mice (splenocytes) cured by the combination of GAd and anti-PD1. Responses against the immunogenic nAgs are shown (*n* = 6–9 mice per group, representative of two independent experiments). **c** nAg-specific T-cell responses measured by IFN-γ ICS in responder and non-responder mice treated with GAd-CT26-31 and anti-PD1. Percentages of IFN-γ^+^ CD4^+^ and CD8^+^ T cells measured at day 30 upon peptides pool re-stimulation are shown. (two-tailed Mann–Whitney *U* test; **p < 0.01, n.s., not significant). **d** Tumor-free mice treated with anti-PD1 and GAd-CT26-31 were challenged with a second CT26 tumor inoculum (*n* = 10, at least three independent experiments). Animals survival after second challenge was monitored until 100 days post re-challenge. (Log-rank test; ***p < 0.001). **e** Tumor growth in tumor bearing mice treated with GAd-CT26-31 and anti-PD1 and depleted for CD4^+^ or CD8^+^ T cells. Data represent at least two independent experiments. (Fisher exact test; *p < 0.05). **f** Number of lung nodules measured in control mice or mice vaccinated with GAd-CT26-31 (*n* = 8 per group from one experiment) and depleted for CD4^+^ or CD8^+^ T cells (two-tailed Mann–Whitney *U* test; **p < 0.01; n.s., not significant)

To improve the effectiveness of GAd vaccine in mice with high tumor burden, the combined treatment of GAd and anti-PD1 was evaluated. Anti-PD1 was chosen as it represents the most widely used CPB and it is approved for many cancer indications. CT26 established tumors responded to anti-PD1 monotherapy in a small fraction of treated animals (15%), by showing complete regression of tumors. Combined treatment of anti-PD1 and GAd-CT26-31 provided remarkable tumor control, causing complete tumor regression in ~50% of mice (Fig. 3a). Surprisingly, combination of GAd-CT26-5 vaccine with anti-PD1 did not improve the cure rate of anti-PD1 alone (Supplementary Fig. 4d).

Animals cured by the combination of GAd-CT26-31 and anti-PD1 showed the induction of a systemic CD4^+^ and CD8^+^ T-cell response comparable to that obtained in naïve mice both in terms of breadth and potency (Fig. 3b). Comparison of nAgs-specific T-cell responses in responder versus non-responder mice showed significantly higher levels of IFNγ^+^ CD8^+^ T cells in the responder group (Fig. 3c). Low levels of a spontaneous immune response against only one of the 31 tested nAgs (nAg#4) was found in animals responding to anti-PD1 treatment alone (Supplementary Fig. 6). Mice that became tumor free after the combined treatment were all protected from a second CT26 tumor challenge, demonstrating effective induction of memory T cells (Fig. 3d). Selective depletion of CD8^+^ T cells completely abrogated the antitumor effect, highlighting the contribution of this lymphocyte population to the treatment efficacy. In contrast, depletion of CD4^+^ T cells did not impact efficacy of the treatment (Fig. 3e). The key role of CD8^+^ T cells was also confirmed in the early therapeutic setting of lung metastases (Fig. 3f).

### Activation of an immune gene signature in responder tumors

To identify functional pathways associated with treatment response or resistance, tumors were collected 17 days from the start of the treatment, when it was possible to discriminate between those in progression (non-responders) from those in regression (responders). DNA and RNA were extracted from tumors of mice belonging to the three treated groups: (i) vaccine alone, (ii) anti-PD1 alone,(iii) combination of anti-PD1 and vaccine and from untreated animals. DNA-exome sequencing and RNASeq on tumors harvested from mice that failed to respond to combined treatment showed that all immunogenic mutations were still present and expressed in non-responders, indicating that lack of response to treatment in those animals was not due to nAgs loss (Supplementary Table 2). Resistance to treatment was not associated with genetic loss of MHC-I or lack of MHC expression (Supplementary Table 3).

**Fig. 4:**
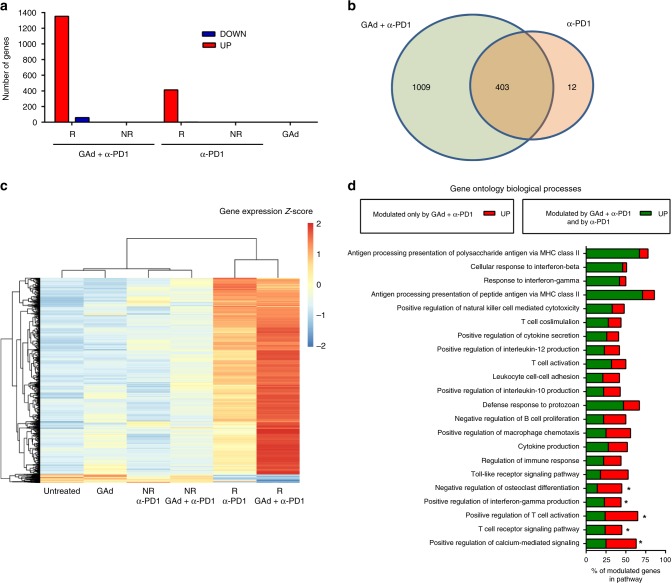
Effective treatments correlate with overexpression of a large number of functionally relevant genes. **a** Number of differentially expressed genes (DEG) (red, upregulated; blue, downregulated) detected by RNAseq on tumors in progression (non-responders, NR) and regression (responders, R) upon anti-PD1 or anti-PD1 and vaccine combination versus untreated tumors (*n* = 3–5/group) (median log2 FC < −1 or >1; Benjamini–Hochberg corrected *p* value < 0.05). **b** Venn diagram showing the overlap of the DEG plotted in panel A between anti-PD1 and combination therapy. **c** Heat map of the 1412 DEG found in responders to the combination therapy compared to untreated for each group of treatment. **d** Gene Ontology (GO) enrichment analysis performed on DEG genes found in responders to GAd and anti-PD1 combination versus untreated. The 22 biological processes with at least the 40% of genes significantly modulated are shown (Bonferroni corrected *p* value < 0.01). Genes upregulated upon combination treatment and anti-PD1 are shown in green, those upregulated only by combination treatment are shown in red. Pathways with a significant difference in percentage of modulated genes in responders to the combo versus responders to anti-PD1 are marked with an asterisk (*p* < 0.05, two-tailed, Fisher test)

Gene expression profiles were also evaluated. Transcriptional profiles of tumors from non-responders in the anti-PD1, vaccine alone, or combination groups were all indistinguishable from those of untreated tumors, consistent with the lack of tumor shrinkage. However, when we compared gene expression of responders, both from anti-PD1 and combination groups, versus untreated tumors, significantly differentially expressed genes (DEG) were observed in tumors from regressors. Although in responder mice treated with combination therapy there were 1353 upregulated and 59 downregulated genes, only 413 genes were upregulated and two downregulated in responder mice treated with anti-PD1 alone (Fig. 4a and Supplementary Data 1), most of whom were shared with the combination group (Fig. 4b). The remaining genes, uniquely upregulated in the combination group, also showed a trend toward induction in mice receiving anti-PD1 only (Fig. 4c), and even the common DEG were more significantly upregulated in the combination group.

Of note, tumors responding to the combination upregulated 15 out of 18 genes belonging to the TIS previously identified in patients responding to anti-PD1 (Supplementary Fig. 7)^27,28^. Gene ontology (GO) enrichment analysis of the 1412 DEG revealed significant changes in pathways related to the immune response. Figure 4d shows biological processes for which at least the 40% of genes were significantly upregulated in combo treated animals. Most of these pathways belong to innate and adaptive immunity activation. Interestingly, five of these biological processes, including regulation of TCR signaling, T-cell activation and interferon gamma production, showed a significant enrichment in the number of genes specifically modulated by the combination treatment compared to anti-PD1 (Fig. 4d and Supplementary Data 2).

### Combined therapy diversifies intratumoral TCR repertoire

**Fig. 5:**
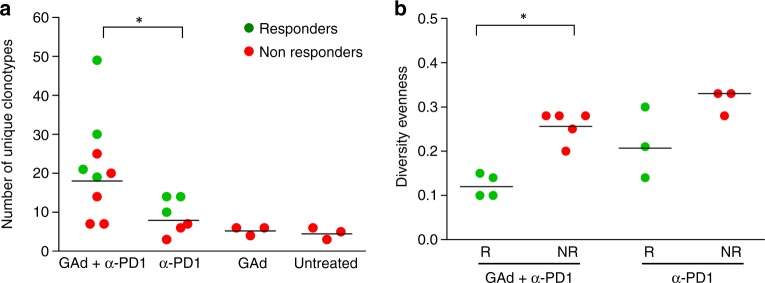
Combined treatment of GAd and anti-PD1 induced enrichment and expansion of TCR-β clonotypes: **a** Number of clonotypes detected in tumors (*n* = 3–5/group) for each group of treatment by analysis of TCR**-**β sequences (green circles are responder tumors, red circles non-responders). **b** Changes in Diversity evenness between responders (R) and non-responders (NR) to combo and anti-PD1 treatment. Diversity evenness was defined as the minimum percentage of CDR3 sequences accounting for 50% of the reads mapped on TCR-β. *p < 0.05, two-tailed Mann–Whitney *U* test

TCR representation was analyzed from RNA-sequencing data of tumors as previously described^33^. As we determined an increased breadth of immune response by IFN-γ ELISpot analysis in mice cured by the combination of GAd-CT26-31 and anti-PD1, we interrogated the transcriptome to evaluate if there were changes in the number of TCR clonotypes in the different treatment groups. The combination treatment indeed resulted in diversification of the intratumoral T-cell repertoire, detected as unique TCR-beta CDR3 sequences. A significant increase of the total number of TCR-beta clonotypes was observed by comparing the combination versus the anti-PD1 monotherapy treatment (Fig. 5a), reflecting a larger T-cell diversity in the former treatment group. Vaccination per se did not result in such enrichment of different clonotypes within the tumor, suggesting lack of expansion of vaccine-induced T cells in the absence of anti-PD1 treatment. A clear difference between responders and non-responders to the combined treatment was found evaluating the TCR-beta CDR3 equality distribution (evenness). Responders to the combination showed lower evenness corresponding to a TCR repertoire dominated by specific T-cell clones. A similar trend, although not significant, was also observed between responders and non-responders to anti-PD1 therapy. This indicates that intratumoral expansion of selected T-cell clones correlates with the response (Fig. 5b).

### Synergy of GAd and CPB is confirmed in a second model

**Fig. 6:**
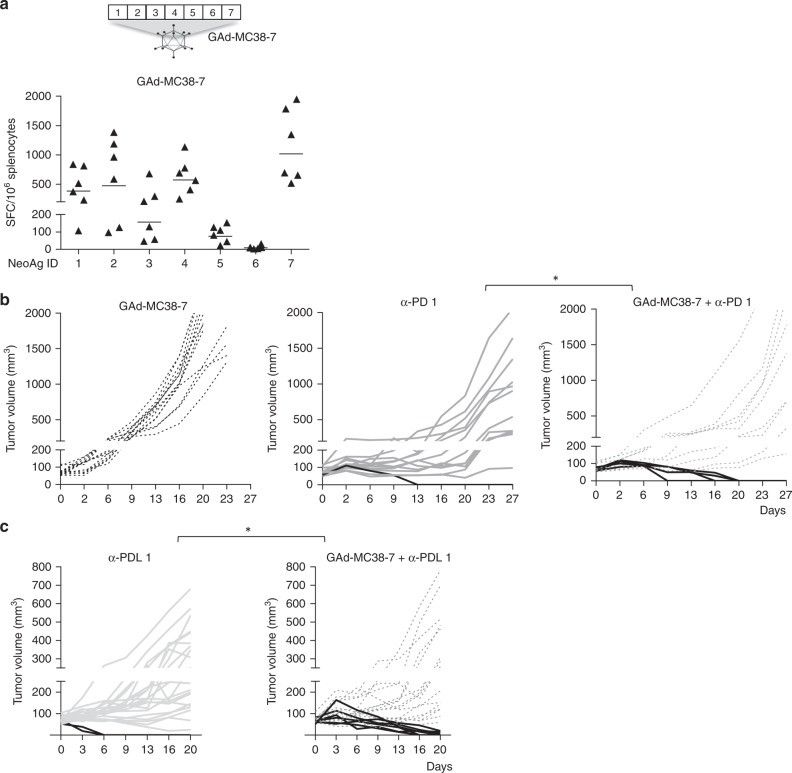
GAd and checkpoint inhibitors also synergize in the MC38 tumor model. **a** C57BL/6 mice (*n* = 6/group) were immunized with a GAd vector encoding seven MC38 neoantigens (GAd-MC38-7). Two weeks post vaccination, immune responses against the seven mutated peptides were measured on splenocytes. Data are representative of two independent experiments with six mice/group for each experiment. SFC = Spot Forming Cells. **b**, **c** Efficacy of GAd-MC38-7 alone and in combination with anti-PD1 **b** or anti-PDL1 **c**. Treatment with GAd vaccine started at day 0, on mice randomized according to tumor volume. Mice showing complete tumor shrinkage post treatment are identified by black lines. Data represent at least two independent experiments (*n* = 8–10/group) (Fisher test. *p <0.05)

The synergy between GAd nAgs-based vaccine and CPB was investigated in the MC38 cell line derived from C57BL/6 murine colon adenocarcinoma. For these experiments, we used seven nAgs previously identified in MC38 cells by mass spectrometry analysis and shown to be able to induce CD8^+^ T-cell responses (Table 2)^5^. All seven mutations were confirmed to be present in tumors of subcutaneously injected mice by exome sequencing (Supplementary Table 4). Each aa change determined by the mutation was flanked upstream and downstream by wild-type aa to generate the nAgs, as described previously for the CT26 nAgs. The seven nAgs were joined head to tail to generate an artificial protein, which was encoded into the GAd vector. A single GAd vaccination of naive mice induced T cells response against six out of seven nAgs (Fig. 6a). All T-cell responses were confirmed to be CD8^+^ by ICS (Supplementary Fig. 8). Importantly, no cross-reactive T-cell responses were detected against the wild-type sequence post vaccination (Supplementary Fig. 2b). Vaccine efficacy was evaluated in animals bearing large MC38 tumors. Vaccination was confirmed to be ineffective as a stand-alone treatment, whereas CPB monotherapy induced regression of established tumors only in a minority of animals (7% anti-PD1; 9% anti-PDL1). In contrast, synergistic activity was demonstrated when the vaccine was combined with either anti-PD1 or anti-PDL1 antibodies with shrinkage of tumors observed in ~ 30% of mice (Fig. 6b, c).

**Table 2:** MC38 neoantigens encoded by GAd-MC38-7 vaccine

ID	Gene symbol	Gene ID	Neoantigen sequence
1	CPNE1	266692	DFTGSNGDPSSP[D/**Y**]SLHYLSPTGVNEY
2	IRGQ	210146	KARDETAALLNSA[G/**V**]LGAAPLFVPPAD
3	AATF	56321	SKLLSFMAPIDHT[A/**T**]MSDDARTELFRS
4	REPS1	19707	GRVLELFRAAQL[P/**A**]NDVVLQIMELCGATR
5	MED12	59024	DIDPSSSVLFE[D/**Y**]MEKPDFSLFSP
6	DPAGT1	13478	EAGQSLVISASIIVFNL[V/**L**]ELEGDYR
7	ADPGK	72141	GIPVHLELASMTN[R/**M**]ELMSSIVHQQVFPT

List of the MC38 neoantigens encoded by GAd-MC38-7 vaccine. The mutated amino acid is in bold next to the wild-type amino acid

## Discussion

Therapeutic cancer vaccines targeting unique patients’ specific mutations are a promising approach to improve the efficacy of CPB therapy by increasing the breadth and potency of nAg-specific T cells. One of the main challenges for the development of therapeutic vaccines is the availability of a delivery system capable of safe and efficient induction of T-cell immunity. Non-human Great Apes Adenoviruses have recently emerged as a safe and powerful genetic vaccine platform in humans. This class of replication-defective adenoviral vectors was tested in over four thousand humans of all ages, showing induction of unprecedented CD8^+^ and CD4^+^ T-cell responses and a good safety profile^20,26,34^.

Here, we investigated for the first time the efficacy of GAd vaccines encoding tumor nAgs. A GAd encoding 31 nAgs (GAd-31), the largest number used so far for nAg-based vaccines, selected from the murine CT26 colon carcinoma cell line, was capable of inducing potent T-cell immunity, with overall >1000 antigen-specific IFN-γ secreting lymphocytes/million splenocytes and the induction of both CD8^+^ and CD4^+^ T lymphocytes. Only a fraction (20%) of predicted nAgs was found to be immunogenic, in line with previously published reports^35^, underlying the current limitations of the neoantigen prediction methods^36^. Indeed, no single method available today allows for a reliable identification of the immunogenic/efficacious neoantigens, making their empirical determination still not feasible in the clinical setting of personalized vaccination. In this scenario, one of the advantages of the GAd vector technology is its ability to accommodate large gene inserts, allowing the targeting of many neoantigens. Therefore, the use of this platform offers the opportunity to overcome the limits of the prediction algorithms. Of note, the 31 nAgs used here do not represent the upper limit of the GAd vectors, as we recently managed to successfully express over 60 nAgs in a single vector (unpublished).

Previous nAgs-based cancer vaccination studies in mice have shown that significant efficacy can be achieved in a model of lung metastases by administering synthetic RNA vaccine encoding five neoantigens, soon after intravenous injection of CT26 tumor cells^7^. To bridge our results with these previously published data, we encoded in GAd the very same set of five nAgs (GAd-5) and observed comparable efficacy in this early therapeutic setting with our GAd-31 vector. However, vaccination with either GAd alone was not capable to reduce disease progression when administered to mice with large established tumors. This lack of efficacy was not owing to an impaired induction of nAgs-specific immune response or inefficient lymphocyte trafficking to the tumor, as GAd vaccine-induced T cells were detected in the tumor post vaccination. Nevertheless, these responses failed to control tumor growth. Transcriptome analysis on tumors harvested 17 days post vaccination from GAd vaccinated and untreated animals did not reveal differences between the two groups either in gene expression profiles or in the number of unique TCR clones present in the tumor, suggesting lack of expansion of vaccine-induced T cells.

Treatment of established tumors with anti-PD1 was able to induce regression in a limited number of mice (15%). Co-treatment with the shorter GAd encoding five previously identified CT26 nAgs^7^ did not change the rate of response. In contrast, combined treatment with the GAd-31 vaccine consistently resulted in a three-fold increase in the number of mice showing complete regression (~ 50%), correlating with increased number of T-cell clones with unique TCR in this stringent setting of high tumor burden. Of note, the GAd-5 shorter construct shared only three immunogenic nAgs with the long GAd-31. Although we did not directly explore whether the difference between the two vaccines is owing to quality or quantity of the encoded neoantigens, our results suggest that the ability to design multi-epitope vaccines may allow to overcome the limits imposed by the inaccuracy of the bioinformatics tools currently available to predict truly immunogenic nAgs.

The antitumor effect of the vaccination was found to be dependent on CD8^+^ T cells in both early and late treatments setting, whereas other studies have shown that vaccination with a single CT26 nAg inducing CD4^+^ T-cell responses reduced the formation of metastases in an early therapeutic setting^37^. Likely, both components of adaptive immune response can play a role at different stages of tumor formation, and the ability of balanced induction of both CD4^+^ and CD8^+^ T cells is an advantageous feature of the GAd vaccination platform.

Despite a significant improvement over mono-therapies, the combined regimen failed to cure about half of the treated mice. Of note, tumor escape was not associated to immunoselection of tumor variants lacking expression of the targeted nAgs. Elucidating the bases for resistance will allow to identify targetable mechanisms to improve the therapeutic efficacy of nAgs vaccination. Tumors in regression after the combined treatments showed upregulation of transcriptional networks linked to T cells activation and effector functions, including 15 out of 18 genes belonging to a TIS described in patients who responded to anti-PD1 immunotherapy^28^. Consistent with immune recognition of the tumor, highly expressed genes included immune-related genes, such as Granzyme B/K associated with cytolytic function, T-cell markers (CD2, CD3D, CD3E, IL2RG), chemokines/chemoattractants (CXCL9, CXCR6, CXCL10), genes involved in antigen presentation (CIITA, H2-T23, and H2-EA-PS) and immunomodulatory molecules such as IDO and LAG3, the last two factors likely being upregulated as a consequence of T-cell activation and IFN-γ signaling to restrain the antitumor immune responses. The pattern of genes modulated in response to combination therapy was also found in anti-PD1 responders, with a global trend towards a more pronounced activation in the combination group, reflecting a partially common effect on immune cells. However, a more-striking difference between the combination and anti-PD1 therapy was found when analyzing the TCR repertoire. Concomitant treatment with GAd vaccine and anti-PD1 increased the TCR diversity, suggesting the broadening of tumor-reactive T cells. Moreover, selective expansion of specific subsets of T-cell clones was found to be associated with efficacy in the combination group, suggesting that the growth of specific immunologically relevant T-cell populations is a key feature for the antitumor activity. The skewing of specific T-cell clonotypes has also been reported in patients responding to immunotherapy^38^, underlying the translational relevance of our observations.

The robustness of our observation was confirmed in a second model by using a GAd vector encoding seven nAgs previously identified in murine MC38 tumor cells by mass spectrometry^5^. Administration of the MC38 GAd vaccine induced potent T-cell responses, with overall >4000 antigen-specific IFN-γ-secreting lymphocytes/million splenocytes, and with six out of seven encoded nAgs being immunogenic. Notably, previous studies using the same nAgs administered as a mixture of synthetic peptides in adjuvants showed that only three out of seven nAgs induced a T-cell response^5^. Moreover, synergy of vaccination with both anti-PD1 or anti-PDL1 treatment was shown in this model by treating animals with large tumors.

GAd vectors represent a novel class of safe, potent, and clinically validated genetic vaccine, which can be manufactured according to a standardized, reliable, and highly reproducible process that is independent from the size and the identity of the encoded antigen. A major obstacle to developing personalized vaccines based on individual nAgs is whether they can be produced in a timeframe that will allow for their administration to patients with advanced tumors. To this end, we have developed a fast process for the assembly of gene strings encoding over 60 unique patient mutanome-specific neoantigens and for the production of personalized GAd vaccines within 6 weeks from the time of patient biopsy for rapid administration to cancer patients.

## Methods

### Mice

Six-week-old female BALB/c or C57BL/6 mice were purchased from Envigo. All day-to-day care was performed by trained mouse house staff at Plaisant, Castel Romano. All experimental procedures were approved by the Italian Ministry of Health (Authorizations 213/2016 PR) and have been done in accordance with the applicable Italian laws (D.L.vo 26/14 and following amendments), the Institutional Animal Care and ethic Committee of CEINGE and Allevamenti Plaisant SRL.

### Cell culture

CT26 (BALB/c mouse undifferentiated colon carcinoma) and MC38 (C57BL/6 mouse colon adenocarcinoma) were purchased from ATCC. Cell lines were cultured in complete RPMI-1640 or Dulbecco's Modified Eagle medium, respectively, supplemented with 10% fetal bovine serum, 2 mm l-glutamine, 1% (v/v) penicillin/streptomycin and maintained at 37 °C in 5% CO_2_. They were not further authenticated but cultured for a limited number of passages. Cell lines were tested for the absence of mycoplasma contamination by PCR.

### Adenoviral vectors production

The coding sequences for CT26-5 and MC38-7 transgenes were purchased as phosphorylated gBlock dsDNA fragments (IDT) and cloned into p-tetOCMV-BGHpA, containg CMV promoter with two TetO repeats and a BGH polyA, previously digested with EcoRV (New England Biolabs). The CDS for CT26-31 was generated by Gibson assembly (New England Biolabs) of two overlapping gBlock sequences (Integrated DNA Technologies) into p-tetOCMV-BGHpA previously digested with EcoRV and Not1 restriction enzymes (New England Biolabs). The expression cassettes were then transferred into pGAd plasmid, containing the E1/E3/E4 deleted in which the E4 is replaced with Ad5 E4 ORF6 of a Great Ape Adenovirus (serotype group C). The transgene cassette was introduced into the E1 deletion by homologous recombination in BJ5183 cells (Agilent). GAds vectors were then produced by transfection of adherent M9 cells (293 cells derivative) with Lipofectamine 2000 (Invitrogen, Thermo Fisher Scientific) and amplification in suspension M9 cells. Vectors were then purified from infected cells by Vivapure Adenopack 20 RT (Sartorius).

### Exome and RNA sequencing

DNA and RNA library construction and NGS tumor samples were performed at Center for Translational Genomics and Bioinformatics (CTGB)—San Raffaele Hospital, and at Genomix4Life S.r.l (Salerno). Genomic DNA was fragmented and used for Illumina library construction. Exonic regions were captured in solution using the Agilent mouse SureSelect All Exon kit 50 Mb. Paired-end sequencing, resulting in 100 bp from each end of fragments, was performed with the Hiseq2000 Genome Analyzer (Illumina) at target coverage of × 120. RNA was fragmented and the sequencing library was prepared using Illumina TruSeq mRNA stranded kit. Sequencing was performed with the Hiseq2000 Genome Analyzer (Illumina) at target depth of 60 mln of paired-end reads. Germline sequences from the respective murine strain were downloaded from SRA (experiment id: ERX391212) and used as control sample for comparison with tumor. Quality control of sequenced reads was performed with FastQC 0.11.5. Reads that aligned to more than one locus with the same mapping score were filtered using Samtools 0.1.19. Somatic SNV were called by using mutect v1.1.17 and varscan2 v2.3.9 with default parameters, by explicitly comparing the tumor sample vs the normal control sample. SNVs detected by at least one of the two variant callers and inducing a missense amino-acid change were mapped on the mm10 Refseq transcriptome by using Annovar. A 25-mer peptide was designed by selecting the mutated amino acid plus 12 wild-type amido acids at both flanking regions. MHC-I and MHC-II-binding predictions were performed by using the consensus method of IEDB 2.17 software. Mutations were prioritized using sequential filtering criteria with a funnel strategy. SNVs with predicted IC50 ≤ 500 nm for MHC-I and percentile rank score threshold ≤ 1 for MHC-II were selected. SNVs with variant allele frequency in tumor ≥ 25% were further selected and finally those expressed with at least one mutated read in RNAseq were retained.

### In vivo tumor growth

For prophylactic experiments, 2 × 10^5^ CT26 cells were s.c. injected into the lower right flank, 2 weeks after immunization. For primary metastases, 1 × 10^5^ CT26 cells were injected i.v. into the tail vein 3 days before vaccination. On day 16, lungs were perfused with India Ink 15%, harvested and fixed in Fekete’s solution. Metastatic colonies on the surface of the lungs were counted using a dissecting microscope. For established tumor setting experiments, 2 × 10^6^ CT26 or 2 × 10^5^ MC38 cells were s.c. injected as aforementioned. Before treatments start (day 0), animals were randomized (tumor size average per group 70–100 mm^3^). Mice were killed as soon as signs of distress or a tumor volume above 2000 mm^3^ occurred. Tumor growth was measured using digital caliper every 3–4 days. Tumor volume was calculated using the formula: 0.5 × length × width^2^, where the length was the longer dimension.

### In vivo treatments

Vaccines were administered via intramuscular injections in the quadriceps by delivering a volume of 50 µl per side at 5 × 10^8^ vp. For efficacy studies, α-hPD-L1-mIgG1 (InvivoGgen, Cat. Number: hpdl1-mab9) and α-mPD1 (BioXcell, clone RMP114, Cat. Number: BE0146) were administered twice a week until day 16 post treatment start. To deplete T-cell subsets, α -mCD8 (BioXcell, clone YTS169.4, Cat. Number: BE0117) and α-mCD4 (BioXcell, clone YTS191, Cat. Number: BE0119) were used. Each antibody was administered i.p. at a dosage of 200 μg.

### Ex vivo immune analysis

IFN-γ ELISpot assays were performed on single-cell suspensions of spleens. MSIP S4510 plates (Millipore, Billerica, MA) were coated with 10 µg/ml of anti-mouse IFN-γ antibody (Cat. Number: CT317-C; U-CyTech) and incubated overnight at 4 °C. After washing and blocking, mouse splenocytes were plated in duplicate at two different cell densities and stimulated overnight with single 25-mer peptides or peptide pool at a final concentration of 1 µg/ml. The peptide diluent dimethyl sulfoxide (Sigma-Aldrich) and concanavalin A (Sigma-Aldrich) were used, respectively, as negative and positive controls. Plates were developed by subsequent incubations with biotinylated anti-mouse IFN-γ antibody (dilution: 1/100; Cat. Number: CT317-D; U-CyTech), conjugated streptavidin–alkaline phosphatase (dilution: 1/2500; Cat. Number 554065; BD Biosciences) and finally with 5-bromo-4-chloro-3-indoyl-phosphate/nitro blue tetrazolium 1-Step solution (Thermo Fisher Scientific). An automated enzyme-linked immunosorbent–spot assay video analysis system automated plate reader was used to analyze plates. ELISpot data were expressed as IFN-γ SFCs per million splenocytes. ELISpot responses were considered positive if all the following conditions occurred: (i) IFN-γ production present in ConA stimulated wells, (ii) the number of spots seen in positive wells was three times the number detected in the mock control wells (dimethyl sulfoxide), (iii) at least 30 specific spots/million splenocytes. Intracellular IFN-γ staining was performed by ON stimulation of splenocytes or isolated TIL with single peptides or peptide pools as antigen at final concentration of 2 µg/ml for each peptide in presence of Golgi plug (catalog 555029, BD Biosciences). Dimethyl sulfoxide (catalog D2650, Sigma-Aldrich) was used as negative control, and phorbolmyristate acetate/ionomycin (Sigma-Aldrich) was used as positive controls. After overnight stimulation, cells were incubated with purified anti-mouse CD16/CD32 (1 µg/10^6^ cells; Cat. Number: 553142; BD Biosciences) and then stained in FACS buffer (phosphate-buffered saline, 1% fetal calf serum) with the following surface antibodies: allophycocyanin anti-mouse CD3e (clone 145-2C11) (dilution: 1/100; Cat. Number: 553066); phycoerythrin anti-mouse CD4 (clone RM4-5) (dilution: 1/100; Cat. Number: 553049) and PerCP anti-mouse CD8a (clone 53–6.7) (dilution: 1/100; Cat. Number: 553036) (all from BD Biosciences). Intracellular staining was performed after treatment with Cytofix/Cytoperm (Cat. Number: 554722) and in the presence of PermWash (Cat. Number: 554723) (both from BD Biosciences) using fluorescein isothiocyanate anti-mouse IFN-γ (clone XMG1.2) (dilution: 1/100; Cat. Number: 554411; BD Biosciences). Stained cells were acquired on a FACS Canto flow cytometer and analyzed using DIVA software (BD Biosciences). At least 20,000 CD8^+^, CD3^+^-gated events were acquired for each sample.

### Tumor biopsy for NGS and RNASeq

Tumors biopsies were collected from treated mice and controls. After washing in RNA later, tumors were cut in pieces of maximum 30 mg, immediately flash-frozen in liquid nitrogen and stored at minus 80 °C until nucleic acid extraction. DNA and RNA extraction is performed by using AllPrep DNA/RNA Mini Kit from QIAGEN, according to the manufacturer’s instructions.

### TILs preparation for IFN-γ ICS

Isolated tumors were first dissociated and digested with collagenase I (Gibco) at 37 °C for 2 hours. Tumor homogenates were depleted from erythrocytes using ACK lysing buffer (Gibco) and filtered through 70-µm cell strainers to generate single-cell suspensions. TIL were isolated from tumor homogenates (Pan T cells isolation kit II, Miltenyi Biotec) and co-cultured with APC from spleen of naive mice in presence of antigen stimuli.

### Gene expression analysis

Differentially expressed genes were estimated by comparing treated versus untreated CT26 tumors using four different methods: Deseq2, EdgeR, limma with Voom correction and NOISeq. A count matrix reporting the number of reads mapping to each gene was determined by using the Rsubread package and gene expression was expressed as Transcripts for Kilobase million (TPM). Genes with <10 total count of mapped reads and expressed with a TPM < 1 were excluded. The Benjamini–Hochberg correction was applied to the list of differentially expressed genes identified by each method. Only genes identified by the consensus of three out of the four methods, with a difference of log2 FC of at least ±1 and a corrected *p* value ≤ 0.05 were retained.

### Biological process enrichment analysis

Enrichment analysis was performed by using the functional annotation chart tool included in DAVID web server using as input the list of differentially expressed genes. Only the GO direct biological processes significantly enriched with a *p* value ≤ 0.05 adjusted with Bonferroni method were considered as significantly enriched. In total, 55 GO biological processes resulted enriched by the genes modulated in tumors that respond to vaccine and anti-PD1 compared with untreated.

### TCR clonality analysis

T-cell receptor β-chain clonality was assessed from the RNAseq data using the MiXCR tool applying the standard parameters reported in the RNAseq workflow of the manual. Raw output of MiXCR (CDR3 sequences and expression of the detected clonotypes) were further analyzed with the R package tcR to obtain summary statistics. The expansion of T-cell clonotypes was determined by using the diversity evenness (DE50) defined as the minimum percentage of unique CDR3 sequences needed to account for the 50% of sequencing reads mapped on TCR genes.

### Statistics

Statistical significance was determined by GraphPad Prism using the nonparametric, two-tailed Mann–Whitney *U* test or as otherwise stated in the figure legend.

### Reporting summary

Further information on research design is available in the Nature Research Reporting Summary linked to this article.

## Supplementary information

Supplementary Information

Description of Additional Supplementary Files

Supplementary Dataset 1

Supplementary Dataset 2

Reporting Summary

## Data Availability

The whole-exome sequencing and RNASeq data that support the finding of this study have been deposited in Sequence Read Archive (SRA) under the accession code PRJNA543001. All the other data of this study are available within the article and its supplementary information files and from the corresponding author upon reasonable request.

## References

[1] Hacohen N, Fritsch EF, Carter TA, Lander ES, Wu CJ (2013). Getting personal with neoantigen-based therapeutic cancer vaccines. Cancer Immunol. Res.

[2] Tran E, Robbins PF, Rosenberg SA (2017). ‘Final common pathway’ of human cancer immunotherapy: targeting random somatic mutations. Nat. Immunol..

[3] Yarchoan M, Hopkins A, Jaffee EM (2017). Tumor mutational burden and response rate to PD-1 inhibition. N. Engl. J. Med..

[4] Rizvi, N. A. et al. Cancer immunology. Mutational landscape determines sensitivity to PD-1 blockade in non-small cell lung cancer. *Science* **348**, 124–128 (2015). 10.1126/science.aaa1348 PMC4993154 25765070

[5] Yadav M (2014). Predicting immunogenic tumour mutations by combining mass spectrometry and exome sequencing. Nature.

[6] Gubin MM (2014). Checkpoint blockade cancer immunotherapy targets tumour-specific mutant antigens. Nature.

[7] Kreiter S (2015). Mutant MHC class II epitopes drive therapeutic immune responses to cancer. Nature.

[8] Carreno BM (2015). Cancer immunotherapy. A dendritic cell vaccine increases the breadth and diversity of melanoma neoantigen-specific T cells. Science.

[9] Ott PA (2017). An immunogenic personal neoantigen vaccine for patients with melanoma. Nature.

[10] Sahin U (2017). Personalized RNA mutanome vaccines mobilize poly-specific therapeutic immunity against cancer. Nature.

[11] Pardoll DM (2012). The blockade of immune checkpoints in cancer immunotherapy. Nat. Rev. Cancer.

[12] Sharma P, Allison JP (2015). The future of immune checkpoint therapy. Science.

[13] Alsaab HO (2017). PD-1 and PD-L1 checkpoint signaling inhibition for cancer immunotherapy: mechanism, combinations, and clinical outcome. Front Pharm..

[14] Topalian SL (2012). Safety, activity, and immune correlates of anti-PD-1 antibody in cancer. N. Engl. J. Med..

[15] Herbst RS (2016). Pembrolizumab versus docetaxel for previously treated, PD-L1-positive, advanced non-small-cell lung cancer (KEYNOTE-010): a randomised controlled trial. Lancet.

[16] Robert C (2015). Pembrolizumab versus Ipilimumab in advanced melanoma. N. Engl. J. Med..

[17] Rosenberg JE (2016). Atezolizumab in patients with locally advanced and metastatic urothelial carcinoma who have progressed following treatment with platinum-based chemotherapy: a single-arm, multicentre, phase 2 trial. Lancet.

[18] Sharma P, Hu-Lieskovan S, Wargo JA, Ribas A (2017). Primary, adaptive, and acquired resistance to cancer immunotherapy. Cell.

[19] Capone S (2013). Development of chimpanzee adenoviruses as vaccine vectors: challenges and successes emerging from clinical trials. Expert Rev. Vaccines.

[20] Barnes E (2012). Novel adenovirus-based vaccines induce broad and sustained T-cell responses to HCV in man. Sci. Transl. Med..

[21] O’Hara GA (2012). Clinical assessment of a recombinant simian adenovirus ChAd63: a potent new vaccine vector. J. Infect. Dis..

[22] Sheehy SH (2012). ChAd63-MVA-vectored blood-stage malaria vaccines targeting MSP1 and AMA1: assessment of efficacy against mosquito bite challenge in humans. Mol. Ther..

[23] Swadling L (2014). A human vaccine strategy based on chimpanzee adenoviral and MVA vectors that primes, boosts, and sustains functional HCV-specific T-cell memory. Sci. Transl. Med..

[24] Ledgerwood JE (2017). Chimpanzee adenovirus vector Ebola Vaccine. N. Engl. J. Med..

[25] Green CA (2015). Chimpanzee adenovirus- and MVA-vectored respiratory syncytial virus vaccine is safe and immunogenic in adults. Sci. Transl. Med..

[26] Borthwick N (2014). Vaccine-elicited human T cells recognizing conserved protein regions inhibit HIV-1. Mol. Ther..

[27] Ayers M (2017). IFN-gamma-related mRNA profile predicts clinical response to PD-1 blockade. J. Clin. Invest.

[28] Danaher P (2018). Pan-cancer adaptive immune resistance as defined by the tumor inflammation signature (TIS): results from The Cancer Genome Atlas (TCGA). J. Immunother. Cancer.

[29] Wang S (2016). Intratumoral injection of a CpG oligonucleotide reverts resistance to PD-1 blockade by expanding multifunctional CD8+ T cells. Proc. Natl. Acad. Sci. USA.

[30] Castle JC (2014). Immunomic, genomic and transcriptomic characterization of CT26 colorectal carcinoma. BMC Genomics.

[31] Bui HH (2005). Automated generation and evaluation of specific MHC binding predictive tools: ARB matrix applications. Immunogenetics.

[32] Huang AY (1996). The immunodominant major histocompatibility complex class I-restricted antigen of a murine colon tumor derives from an endogenous retroviral gene product. Proc. Natl. Acad. Sci. USA.

[33] Bolotin DA (2017). Antigen receptor repertoire profiling from RNA-seq data. Nat. Biotechnol..

[34] Green CA (2015). Safety and immunogenicity of novel respiratory syncytial virus (RSV) vaccines based on the RSV viral proteins F, N and M2-1 encoded by simian adenovirus (PanAd3-RSV) and MVA (MVA-RSV); protocol for an open-label, dose-escalation, single-centre, phase 1 clinical trial in healthy adults. BMJ Open.

[35] Castle JC (2012). Exploiting the mutanome for tumor vaccination. Cancer Res..

[36] The problem with neoantigen prediction. *Nat. Biotechnol.* **35**, 97 (2017). 10.1038/nbt.3800. 10.1038/nbt.3800 28178261

[37] Kranz LM (2016). Systemic RNA delivery to dendritic cells exploits antiviral defence for cancer immunotherapy. Nature.

[38] Riaz N (2017). Tumor and microenvironment evolution during Immunotherapy with Nivolumab. Cell.

