# Supplementary material for: Adenoviral vaccine targeting multiple neoantigens as strategy to eradicate large tumors combined with checkpoint blockade
Source: Nat Commun. 2019 Jun 19;10:2688. doi: 10.1038/s41467-019-10594-2 (PMC6584502; doi:10.1038/s41467-019-10594-2)
Supplement: Supplementary file 1 — Supplementary Information [file 41467_2019_10594_MOESM1_ESM.pdf]

Supplementary Information for

Adenoviral vaccine targeting multiple neoantigens as strategy to eradicate large tumors combined with checkpoint blockade

D'Alise et al

This PDF file includes:

Supplementary Figures 1-9

Supplementary Tables 1-4

Supplementary Figures

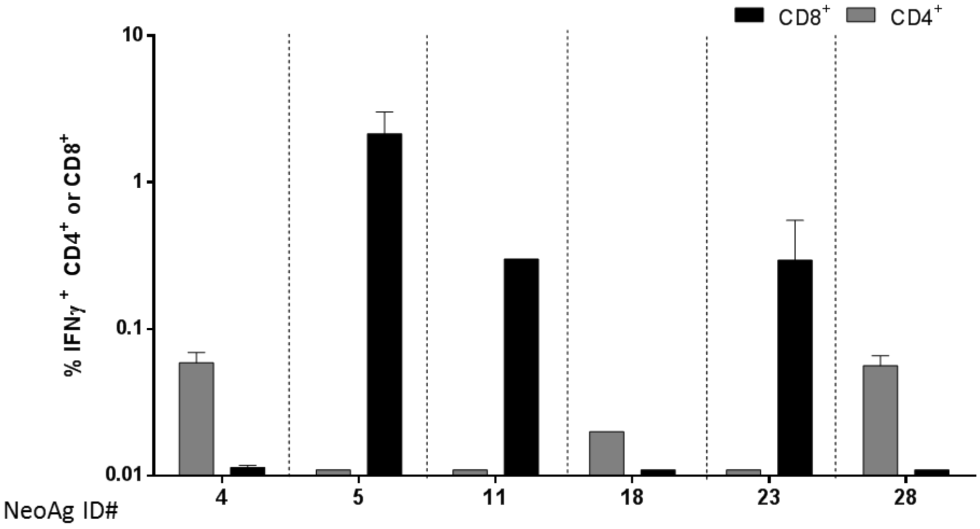

Supplementary Figure 1. Vaccine-encoded immunogenic neoantigens induce both CD8$^+$ and CD4$^+$ T cell responses. Characterization of T cell subtypes induced by immunogenic nAgs after GAd-CT26-31 vaccination evaluated by intracellular cytokine staining (ICS) and fluorescence-activated cell sorting (FACS). 3 weeks post vaccination splenocytes were stimulated with individual immunogenic peptides. Data (means ± SEM) show the percentages of IFN-γ $^+$ CD4$^+$ and CD8$^+$ T cells and are representative of 2 experiments.

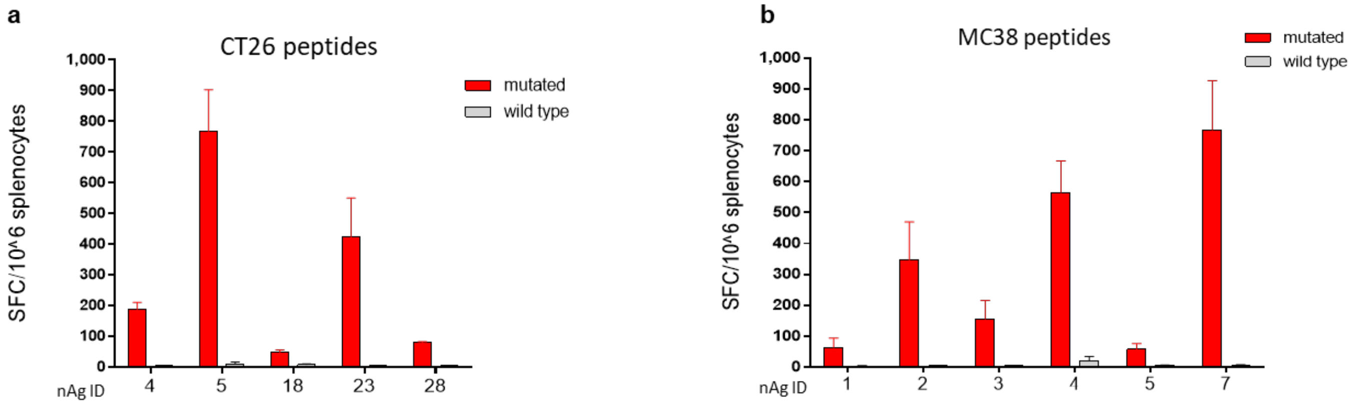

Supplementary Figure 2. GAd vaccination induces T cell immunity against mutated but not wild type peptides. T cell responses measured in mice vaccinated with GAd vectors encoding (**a**) CT26 or (**b**) MC38 neoantigens. Responses against the most immunogenic mutated peptides (red bars) or the wild type peptides (gray) are shown (n=4/group, Mean ± SEM). Vaccination was performed with GAd-CT26-31 or GAd-MC38-7, respectively in a and b.

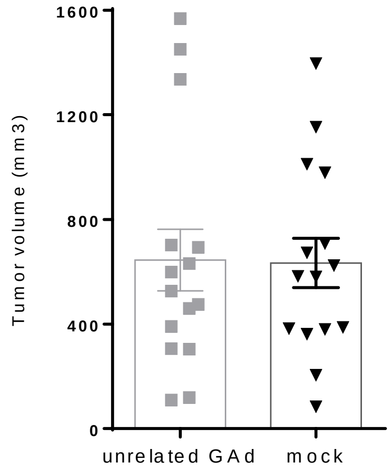

Supplementary Figure 3. Mice vaccinated with unrelated GAd develop tumors similarly to mock controls. Mice (n=15/group) were vaccinated with a GAd encoding unrelated nAgs or left untreated (mock). 2 weeks after immunization, CT26 cells were injected s.c. and tumor growth was monitored over time. Tumor volumes measured 25 days post cells' inoculation in unrelated GAd treated mice versus control (mock) mice are shown. Data are representative of 2 experiments.

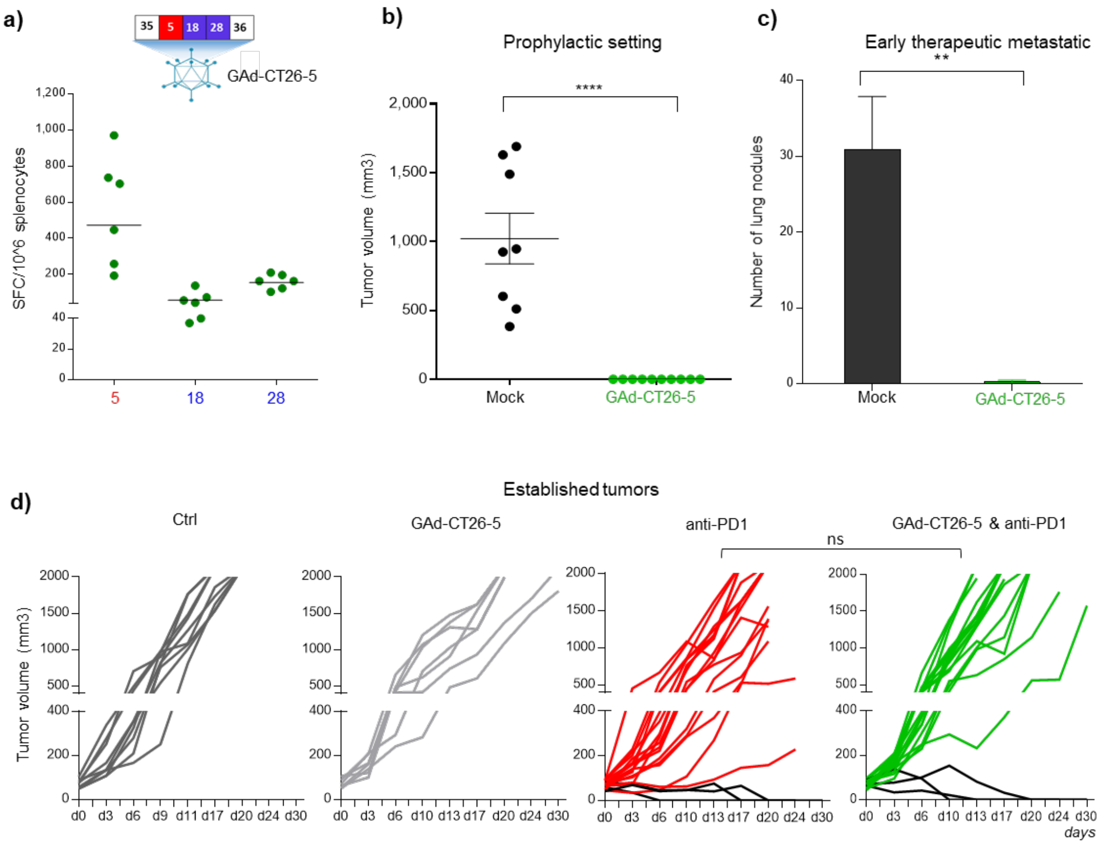

Supplementary Figure 4. Efficacy of GAd vaccine encoding 5 CT26 neoantigens. a T cell responses were measured by IFN-γ ELISpot on splenocytes of naïve mice 3 weeks post immunization with $5x10^8$vp of GAd-CT26-5 (n=6). Responses against individual nAgs peptides found immunogenic are shown; nAgs ID is in red for nAgs inducing $CD8^+$ T cell responses or in blue for nAgs inducing $CD4^+$ T cell responses. **b** Mice (n=8-10/group) were vaccinated with GAd-CT26-5. 2 weeks after immunization, CT26 cells were injected s.c. and tumor growth was monitored over time. Tumor volumes measured 28 days post cells' inoculation in GAd-CT26-5 versus control (mock) mice are shown. **c** Mice (n=8-10/group) were inoculated i.v. with CT26 cells (day 0) and left untreated (mock) or vaccinated with GAd-CT26-5 at day 3. The number of lung nodules counted at day 16 is shown. **d** Efficacy of GAd-CT26-5, anti-PD1 or combination of GAd-CT26-5 with anti-PD1 in tumor bearing mice randomized at day 0 according to tumor volume (mean $70\text{-}100mm^3$). The vaccine was administered at day 0 (i.m.), while anti-PD1 was given twice per week until day 16 (i.p.). Shown is tumor growth in individual mice over time. Black curves represent responder mice. $^{**}P < 0.01$, and $^{****}P < 0.0001$, by 2-tailed Mann-Whitney U test. All data are representative of at least 2-3 independent experiments.

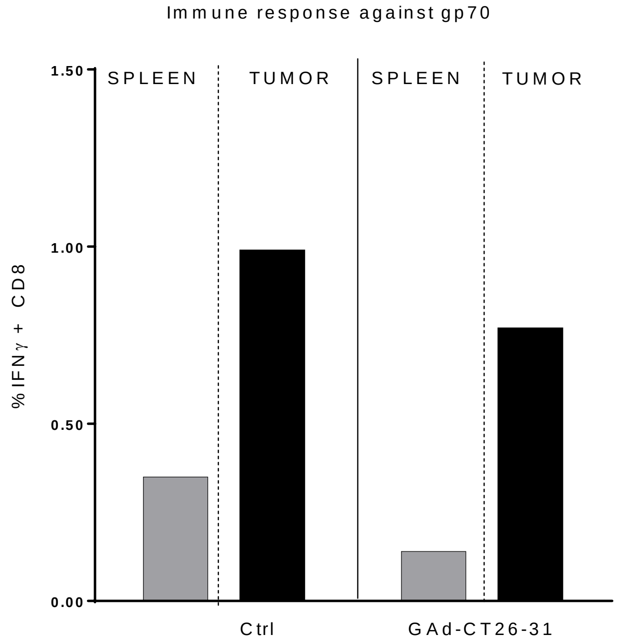

Supplementary Figure 5. $CD8^+$ T cells against gp70-AH1 are present in untreated and vaccinated mice. Percentages of IFN-γ^+ $CD8^+$T cells gp-70 specific measured by ICS on pool of splenocytes and TIL from untreated or GAd-CT26-31 vaccinated mice (n=4). Data are representative of 2 independent experiments.

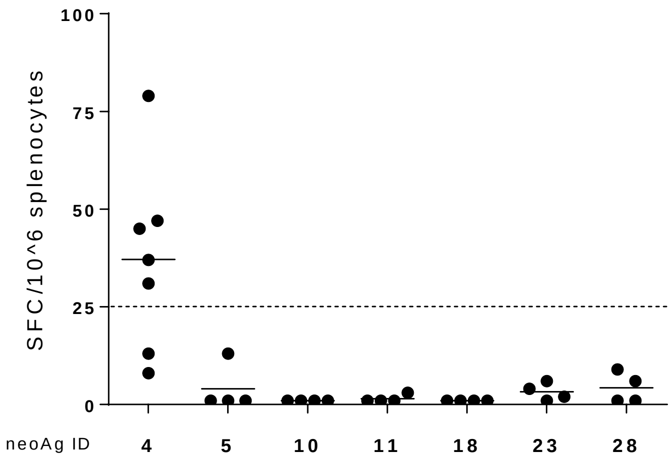

Supplementary Figure 6. Immune response in mice responding to anti-PD1 monotherapy. nAg-specific T cell responses quantified by IFN-γ ELISpot in responder mice (n=4-7/group) treated with anti-PD1. Responses against the seven immunogenic nAgs are shown. Dashed line represents the threshold for a positive response. Data are representative of 3 independent experiments.

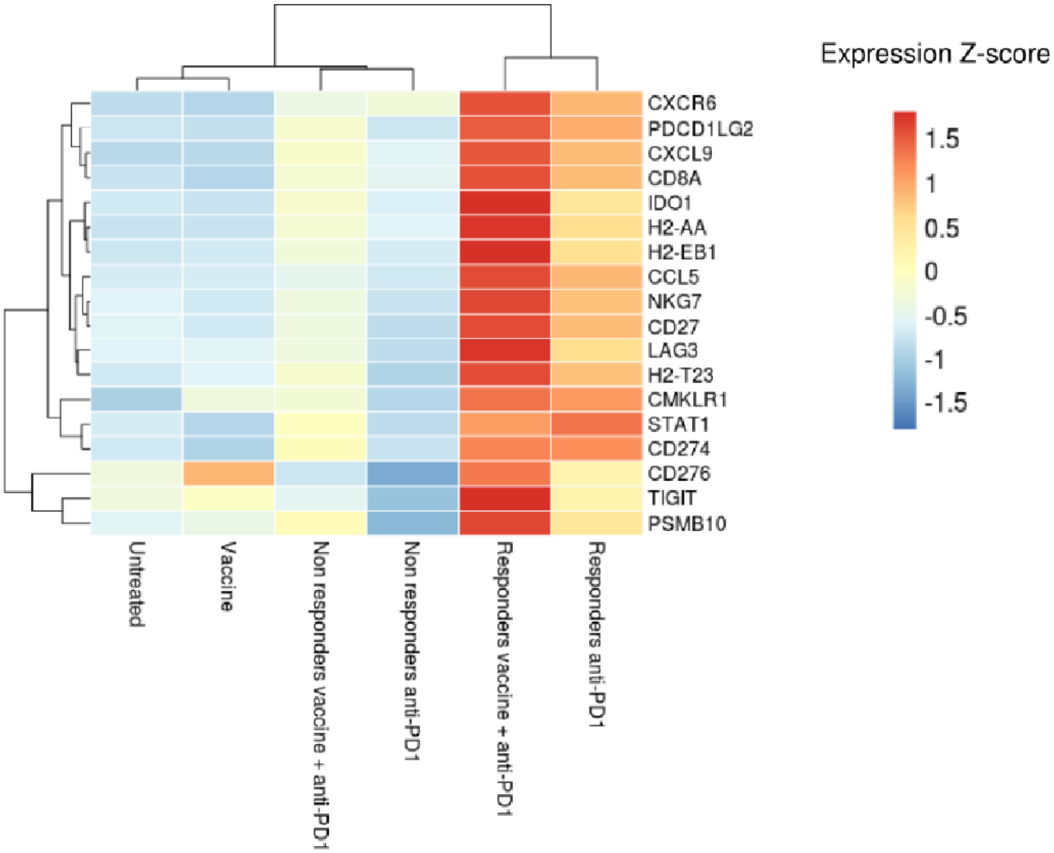

Supplementary Figure 7. Genes belonging to Tumor Inflammation Signature are significantly modulated by combined treatment. Heat map showing the mean expression of the 18 genes belonging to the TIS signature in the 6 categories of mouse tumor samples. The human genes HLA-DQA, HLA-E, HLA-DRB5 included in the TIS have been substituted with the closest mouse orthologs HLA-AA, H2-T23, H2-EB1.

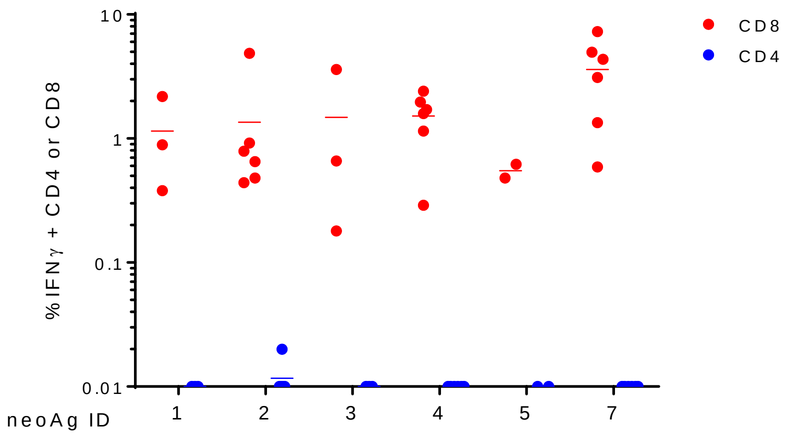

Supplementary Figure 8. The six immunogenic nAgs encoded by GAd-MC38-7 are CD8+ T cell specific. The quality of induced T cell responses (CD4, blue, or CD8, red) was assessed by IFN-γ ICS in naïve mice (n=3-6). Responses against the six immunogenic nAgs are shown.

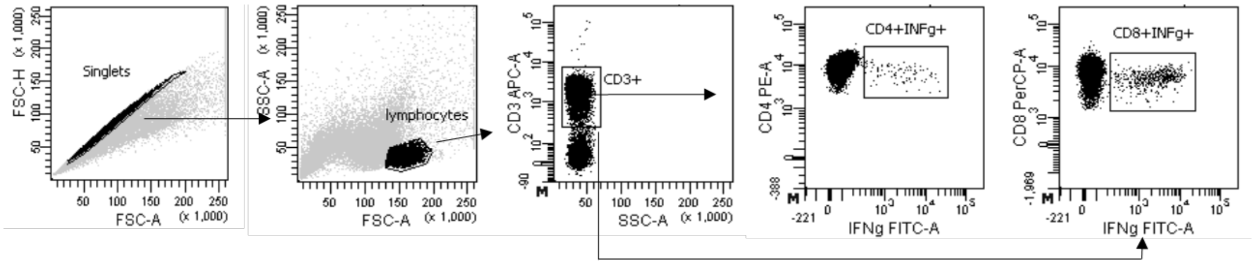

Supplementary Figure 9. Gating strategy used to analyze the frequency of vaccine-induced nAg-T cells producing IFN-γ. CD4+ and CD8+ T cells producing IFN-γ upon peptide restimulation were quantified according this gating strategy for the data presented in Fig.1c, Fig.2d, Fig.3c.

Supplementary Tables

Supplementary Table 1. CT26 neoantigens encoded by GAd-CT26-5 vaccine

nAg ID	Gene Symbol	Gene ID	NEOANTIGEN SEQUENCE
35	SLC4A3	20536	LLPFYPPDEALE**[**T/**I]**GLELNSSALPPT
5	E2f8	108961	ILPQAPSGPSYA**[**I/**T]**YLQPAQAQMLTP
18	Slc20a1	20515	KPLRRNNSYTSY**[**T/**I]**MAICGMPLDSFR
28	Dhx35	71715	VIQTSKYYMRDV**[**T/**I]**AIESAWLLELAP
36	AGXT2L2	72947	HIHRAGGLFVAD**[**E/**A]**IQVGFGRIGKHF

List of the CT26 neoantigens encoded by GAd-CT26-5 vaccine; mutation is indicated in bold next to the wild-type amino acid sequence.

Supplementary Table 2. Mutation allele frequency and expression of CT26 immunogenic mutations in tumors of responders (R) and non responders (NR) mice treated with GAd-CT26-31 vaccine and anti-PD1.

Mutation Allele Frequency (%) in tumor DNA

nAg ID	GENE	NR 1	NR 2	NR 3	NR 4	NR 5	R 1	R 2	R 3	R 4
4	Aldh18a1	60	57	59	57	63	38	26	35	37
5	E2f8	37	36	52	45	38	32	22	29	33
10	Ndc1	69	68	83	65	70	34	23	46	47
11	Glud1	77	81	79	71	76	42	35	50	57
18	Slc20a1	38	39	46	39	72	18	16	26	38
23	Mtch1	36	39	34	43	46	34	-	37	44
28	Dhx35	33	33	41	33	42	20	18	25	22

Number of mutated/wt reads in tumor RNA

nAg ID	GENE	NR 1	NR 2	NR 3	NR 4	NR 5	R 1	R 2	R 3	R 4
4	Aldh18a1	64/31	28/21	43/21	103/57	47/41	41/25	26/29	31/19	54/47
5	E2f8	56/86	33/46	46/54	79/102	44/80	28/42	45/60	26/29	47/58
10	Ndc1	13/8	12/0	22/4	32/6	25/6	15/4	29/8	17/4	14/1
11	Glud1	537/84	445/50	424/80	695/112	493/95	355/146	436/272	333/130	448/133
18	Slc20a1	179/184	115/121	177/193	150/152	164/172	66/123	85/140	74/125	146/131
23	Mtch1	155/270	155/195	145/270	219/365	188/295	151/185	-	172/362	159/302
28	Dhx35	8/15	8/7	10/13	14/18	10/12	3/21	5/23	9/18	5/12

Supplementary Table 3. Expression (TPM) of MHC-I and antigen processing related genes in CT26 tumors of responders (R) and non responders (NR) mice treated with GAd-CT26-31 vaccine and anti-PD1.

GENE	NR 1	NR 2	NR 3	NR 4	NR 5	R 1	R 2	R 3	R 4
H2-K	689.8	615.2	647.8	808.4	747.0	702.0	880.9	874.5	815.4
B2M	8090.9	7225.7	7134.4	6355.7	6064.7	7793.0	8598.1	7004.0	6559.5
TAP1	226.3	149.0	188.3	224.0	165.7	229.5	266.6	245.5	307.6
ERP57	512.5	489.7	544.8	554.8	572.2	598.1	611.7	575.1	546.6
ERAP1	96.6	71.9	88.6	90.5	83.7	87.7	88.9	63.7	88.8

Supplementary Table 4. Mutation Allele Frequency (%) and Expression of nAgs mutations encoded by GAd-MC38-7 in MC38 cell line.

**nAg ID**	**Gene**	**Mutation Allele Frequency (%)**	**Number of reads** (mutated/wt)
1	CPNE1	34	165/63
2	IRGQ	40	1/2
3	AATF	40	85/106
4	REPS1	17	17/67
5	MED12	59	8/1
6	DPAGT1	46	52/35
7	ADPGK	23	13/46
