# Supplementary material for: Adenoviral vaccine targeting multiple neoantigens as strategy to eradicate large tumors combined with checkpoint blockade
Source: Nat Commun. 2019 Jun 19;10:2688. doi: 10.1038/s41467-019-10594-2 (PMC6584502; doi:10.1038/s41467-019-10594-2)
Supplement: Supplementary file 2 — Description of Additional Supplementary Files [file 41467_2019_10594_MOESM2_ESM.docx]

**Title: Supplementary Data 1
Description:** List of differentially expressed genes (DEG) found in tumors of responder mice treated with anti-PD1 and GAd-CT26-31 versus untreated tumors.

**Title: Supplementary Data 2
Description:** List of GO Biological Processes enriched in DEG genes found in tumors of responder mice treated with anti-PD1 and GAd-CT26-31 versus untreated tumors. The top 22 Biological processes with at least the 40% of genes significantly modulated are highlighted in bold (Bonferroni corrected p-value < 0.01).
