# Supplementary material for: Adenoviral vaccine targeting multiple neoantigens as strategy to eradicate large tumors combined with checkpoint blockade
Source: Nat Commun. 2019 Jun 19;10:2688. doi: 10.1038/s41467-019-10594-2 (PMC6584502; doi:10.1038/s41467-019-10594-2)
Supplement: Supplementary file 5 — Reporting Summary [file 41467_2019_10594_MOESM5_ESM.pdf]

nature research

Corresponding author(s): NCOMMS-18-22411B

Last updated by author(s): 20-5-2019

Reporting Summary

Nature Research wishes to improve the reproducibility of the work that we publish. This form provides structure for consistency and transparency in reporting. For further information on Nature Research policies, see Authors & Referees and the Editorial Policy Checklist.

Statistics

For all statistical analyses, confirm that the following items are present in the figure legend, table legend, main text, or Methods section.

n/a	Confirmed	
☐	☒	The exact sample size (*n*) for each experimental group/condition, given as a discrete number and unit of measurement
☐	☒	A statement on whether measurements were taken from distinct samples or whether the same sample was measured repeatedly
☐	☒	The statistical test(s) used AND whether they are one- or two-sided<br>*Only common tests should be described solely by name; describe more complex techniques in the Methods section.*
☒	☐	A description of all covariates tested
☐	☒	A description of any assumptions or corrections, such as tests of normality and adjustment for multiple comparisons
☐	☒	A full description of the statistical parameters including central tendency (e.g. means) or other basic estimates (e.g. regression coefficient) AND variation (e.g. standard deviation) or associated estimates of uncertainty (e.g. confidence intervals)
☐	☒	For null hypothesis testing, the test statistic (e.g. *F*, *t*, *r*) with confidence intervals, effect sizes, degrees of freedom and *P* value noted<br>*Give P values as exact values whenever suitable.*
☒	☐	For Bayesian analysis, information on the choice of priors and Markov chain Monte Carlo settings
☒	☐	For hierarchical and complex designs, identification of the appropriate level for tests and full reporting of outcomes
☒	☐	Estimates of effect sizes (e.g. Cohen's *d*, Pearson's *r*), indicating how they were calculated

Our web collection on statistics for biologists contains articles on many of the points above.

Software and code

Policy information about availability of computer code

Data collection	NA
Data analysis	NA

For manuscripts utilizing custom algorithms or software that are central to the research but not yet described in published literature, software must be made available to editors/reviewers. We strongly encourage code deposition in a community repository (e.g. GitHub). See the Nature Research guidelines for submitting code & software for further information.

Data

Policy information about availability of data

All manuscripts must include a data availability statement. This statement should provide the following information, where applicable:

- Accession codes, unique identifiers, or web links for publicly available datasets
- A list of figures that have associated raw data
- A description of any restrictions on data availability

Data supporting the finding of this study have been deposited in Sequence Read Archive (SRA) under the accession code PRJNA543001 [https://www.ncbi.nlm.nih.gov/bioproject/PRJNA543001]. All the other data of this study are available within the article and its supplementary information files and from the corresponding author upon reasonable request.

Field-specific reporting

Please select the one below that is the best fit for your research. If you are not sure, read the appropriate sections before making your selection.

☒ Life sciences ☐ Behavioural & social sciences ☐ Ecological, evolutionary & environmental sciences

For a reference copy of the document with all sections, see nature.com/documents/nr-reporting-summary-flat.pdf

Life sciences study design

All studies must disclose on these points even when the disclosure is negative.

Sample size	No statistical methods were used to predetermine sample size.
Data exclusions	No data exclusion was performed.
Replication	The experimental findings were reliably reproduced.
Randomization	Mice were chosen at random for each group prior to tumor cells inoculation
Blinding	Blinding not performed, except for tumor calibrations performed by staff blinded to the treatment assignment.

Reporting for specific materials, systems and methods

We require information from authors about some types of materials, experimental systems and methods used in many studies. Here, indicate whether each material, system or method listed is relevant to your study. If you are not sure if a list item applies to your research, read the appropriate section before selecting a response.

Materials & experimental systems

n/a	Involved in the study	
☐	☒	Antibodies
☐	☒	Eukaryotic cell lines
☒	☐	Palaeontology
☐	☒	Animals and other organisms
☒	☐	Human research participants
☒	☐	Clinical data

Methods

n/a	Involved in the study	
☒	☐	ChIP-seq
☐	☒	Flow cytometry
☒	☐	MRI-based neuroimaging

Antibodies

Antibodies used	anti-mouse CD16/CD32 (clone 2.4G2; Cat. Number: 553142, BD Biosciences); APC anti-mouse CD3e (clone 145-2C11; Cat. Number: 553066, BD Biosciences); PE anti-mouse CD4 (clone RM4-5; Cat. Number: 553049, BD Biosciences) ; PerCP anti-mouse CD8a (clone 53–6.7; Cat. Number: 553036, BD Biosciences); FITC anti-mouse IFN-γ (clone XMG1.2; Cat. Number: 554411; BD Biosciences).
Validation	The validation statements can be found on the manufacturer's website

Eukaryotic cell lines

Policy information about cell lines

Cell line source(s)	ATCC
Authentication	no further authentication performed
Mycoplasma contamination	Cells were tested for Mycoplasma and only Mycoplasma free cells were used
Commonly misidentified lines (See ICLAC register)	NA

Animals and other organisms

Policy information about studies involving animals; ARRIVE guidelines recommended for reporting animal research

Laboratory animals	Six-week-old female BALB/c or C57BL/6 mice.
Wild animals	The study didn't involve wild animal.
Field-collected samples	The study didn't involve samples collected from field.
Ethics oversight	The Ethics Committee of the Italian Ministry of Health approved all experiments in this study.

Note that full information on the approval of the study protocol must also be provided in the manuscript.

Flow Cytometry

Plots

Confirm that:

☒ The axis labels state the marker and fluorochrome used (e.g. CD4-FITC).

☒ The axis scales are clearly visible. Include numbers along axes only for bottom left plot of group (a 'group' is an analysis of identical markers).

☒ All plots are contour plots with outliers or pseudocolor plots.

☒ A numerical value for number of cells or percentage (with statistics) is provided.

Methodology

Sample preparation	Mice splenocytes were stimulated at 37 °C in 5% CO2 for 15–20 hours using peptides as antigen in presence of Golgi plug (BD Biosciences) and Brefeldin A. After overnight stimulation, cells were incubated with purified anti-mouse CD16/CD32 clone 2.4G2 (Fc block: BD Biosciences) and then stained in FACS buffer (phosphate-buffered saline, 1% fetal calf serum) with the indicated surface antibodies. Intracellular staining was performed after treatment with Cytofix/Cytoperm and in the presence of PermWash (BD Biosciences). Stained cells were washed twice and resuspended in FACS buffer before analysis.
Instrument	FACS Canto
Software	DIVA software (BD Biosciences).
Cell population abundance	At least 30,000 CD8+, CD3+ gated events were acquired for sample.
Gating strategy	FSC-A/SSC-A was used for gating on lymphocytes .The gating strategy is indicated in Supplementary figures.

☒ Tick this box to confirm that a figure exemplifying the gating strategy is provided in the Supplementary Information.
